# Cross-cultural adaptation and psychometric properties of the Indonesian version of the short acculturation scale

**DOI:** 10.1007/s44202-025-00429-1

**Published:** 2025-09-30

**Authors:** Amirah Zafirah Zaini, Mahmoud Danaee, Tharani Loganathan, Sally Hargreaves, Hazreen Abdul Majid

**Affiliations:** 1 Department of Social and Preventive Medicine, Faculty of Medicine, Universiti Malaya, Kuala Lumpur, 50603 Malaysia; 2 Centre for Epidemiology and Evidence-Based Practice, Department of Social and Preventive Medicine, Faculty of Medicine, Universiti Malaya, Kuala Lumpur, 50603 Malaysia; 3 The Migrant Research Group and The Consortium for Migrant Worker Health, Institute for Infection and Immunity, City St George’s, University of London, London, SW17 0RE UK; 4 School of Health and Rehabilitation Sciences, Health Sciences University, Parkwood Campus, Bournemouth, BH5 2DF UK; 5 Centre for Population Health, Department of Social and Preventive Medicine, Faculty of Medicine, Universiti Malaya, Kuala Lumpur, 50603 Malaysia

**Keywords:** Short acculturation scale, Cross-cultural adaptation, Indonesian language, Psychometric validation

## Abstract

**Background:**

Acculturation plays a crucial role in shaping the health and social outcomes of migrant populations. Despite being one of the largest labour migrant groups in Malaysia, Indonesian migrant workers’ acculturation experiences remain understudied, particularly through culturally appropriate instruments. This study cross-culturally adapted and validated the Short Acculturation Scale for use among Indonesian migrant workers in Malaysia.

**Methods:**

The adaptation and validation of the Short Acculturation Scale involved expert review for content relevance and clarity, forward-backward translation by qualified translators, pilot testing with a sample of the target population, and psychometric evaluation, comprising construct validity and reliability analysis.

**Results:**

Eight of the original 12 items demonstrated excellent content validity, and was successfully translated into Indonesian with semantic and conceptual equivalence. A total of 135 Indonesian migrant workers participated in the pilot testing. Exploratory factor analysis supported a three-factor structure, and internal consistency across subscales was acceptable (Cronbach’s alpha = 0.679–0.816; Spearman-Brown = 0.732). Test-retest reliability showed excellent stability (ICC = 0.991-1.000, *p* < 0.001).

**Conclusions:**

The adapted 8-item Indonesian version of the Short Acculturation Scale demonstrated acceptable construct validity and reliability, supporting its application among Indonesian migrant workers in Malaysia.

**Supplementary Information:**

The online version contains supplementary material available at 10.1007/s44202-025-00429-1.

## Background

Acculturation refers to the process by which immigrants adjust their attitudes, values, and behaviours through interaction with a new culture [1, 2]. Acculturation has a substantial impact on the health outcomes of migrant populations, influenced by factors such as length of residence, proficiency in local languages, social interaction, and familiarity with host cultures [1, 3]. While some studies link acculturation to positive health outcomes, such as improved healthcare access [4–8], others associate it with adverse effects, including cultural stress [9] and unhealthy behavioural changes [10, 11]. These mixed findings underscore the complexity of acculturation and the need for culturally appropriate and psychometrically sound instruments to measure it accurately across diverse migrant populations.

The Short Acculturation Scale (SAS) stands out as one the most widely used instruments among more than 50 existing acculturation measures [3]. Developed by Marin and colleagues [12], the SAS is a 12-item self-report instrument assessing acculturation across three subscales: (a) language use in everyday activities (Subscale 1), including reading, thinking, and speaking, (b) language preferences in media consumption (Subscale 2), and (c) ethnic preferences in social settings (Subscale 3). Scores are calculated as a mean across items, with scores below 2.99 indicating lower acculturation (greater orientation toward the culture of origin), and scores above 2.99 indicating higher acculturation (greater integration into the host culture) [13]. The SAS has been adapted and validated across various migrant groups, including Filipino Americans, Chinese Americans, Korean immigrants in the United States, Pakistani women in Hong Kong, and multicultural populations in Singapore [13–17].

Malaysia is a major migration hub in Southeast Asia, with non-citizens comprising an estimated 10% of the national population in 2024 [18]. The majority of these migrants are low- and semi-skilled labour migrants, with approximately 2.5 million registered workers employed primarily in the manufacturing, construction, and service sectors [19, 20]. Indonesians represent one of the three largest migrant worker communities in the country, accounting for about 24% of the total migrant workforce [19]. Migrant workers in Malaysia face a range of health challenges, including exposure to infectious diseases, occupational injuries, and psychological distress [21–26]. They also face structural and legal barriers to accessing healthcare services [27–30].

Despite the significant presence of Indonesian migrant workers in Malaysia, limited research has explored their acculturation experiences and associated health implications, particularly using validated, culturally appropriate measurement tools. While the SAS has been adapted and validated across various international contexts [13–17], it has not yet been tested for suitability in this population. Beyond its widespread application and strong psychometric performance, the SAS was chosen for its comprehensive theoretical foundation and practical advantages. The SAS allows for a quick yet multidimensional assessment of acculturation by capturing language use in everyday activities and media consumption, as well as social preferences—dimensions that are particularly relevant to the lived experiences of labour migrants. Moreover, its concise structure and simple wording make it especially appropriate for Indonesian migrant workers, many of whom have limited formal education [23, 31–33].

This study aims to cross-culturally adapt and translate the SAS into Indonesian, and to evaluate its validity and reliability for use among Indonesian migrant workers in Malaysia.

## Methods

**Fig. 1:**
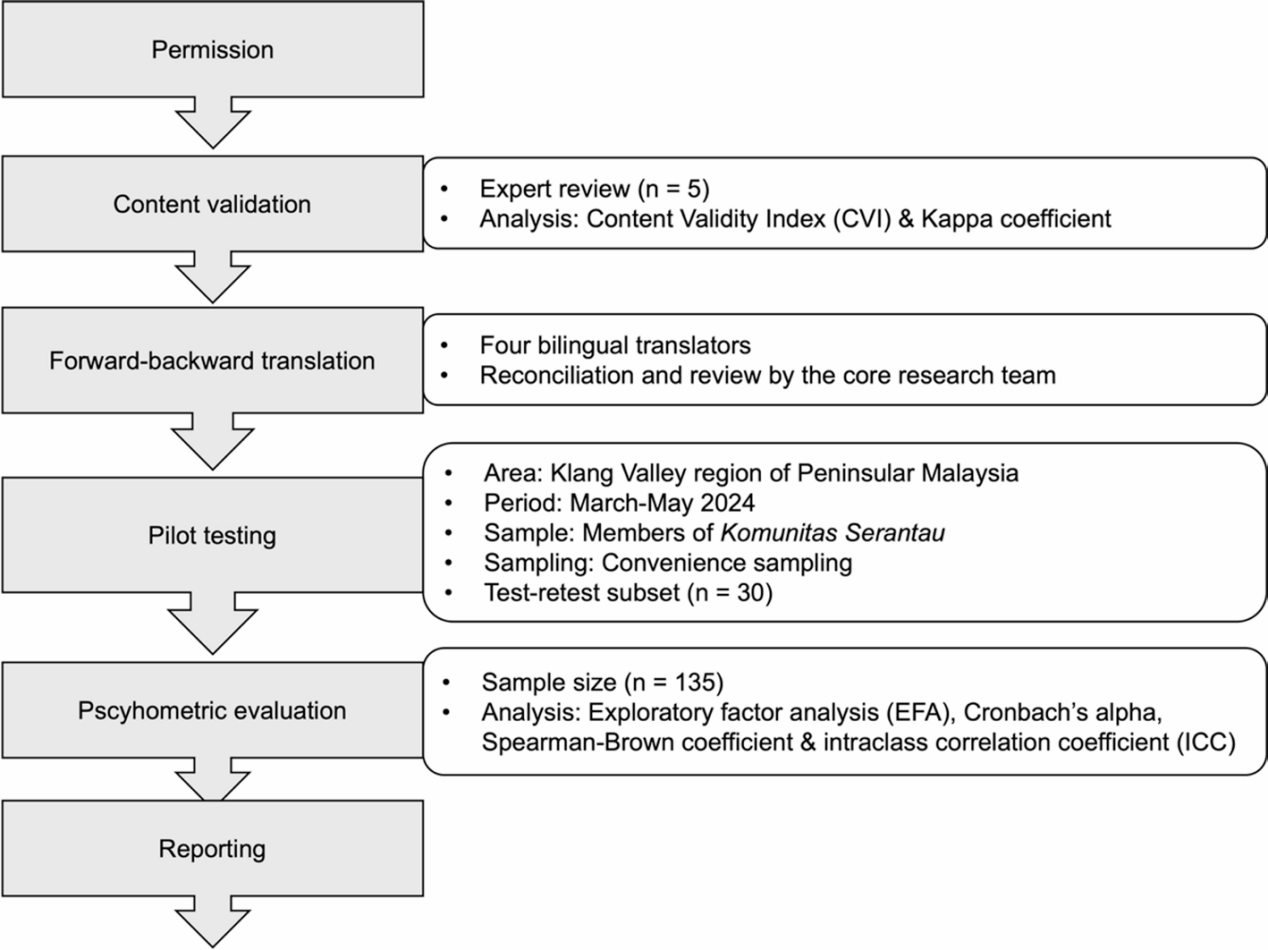
Overview of the study process and methodology

This study was conducted in accordance with the International Test Commission (ITC) Guidelines for Translating and Adapting Tests, which outline six stages: pre-condition, test development, confirmation, administration, scoring and interpretation, and documentation [34]. Permission to adapt the SAS was obtained from the original authors [12], and ethical approval was granted by the Universiti Malaya Research Ethics Committee (Non-Medical) (Reference number: UM.TNC2/UMREC_3136) prior to the study. The adaptation and validation process involved expert review for content relevance and clarity, forward-backward translation by qualified translators, pilot testing with a sample of the target population, psychometric evaluation, and documentation of the procedures (Fig. 1).

### Content validation

The SAS underwent content relevance and clarity assessment by a panel of five experts, comprising one public health specialist, one leader of an Indonesian migrant workers’ community-based organisation, two academics specialising in anthropology and sociology, and one scholar in the field of labour migration. Each expert independently evaluated the relevance and clarity of the items using a 4-point Likert scale, ranging from “1 = not relevant/clear” to “4 = highly relevant/clear”. Experts were also encouraged to provide qualitative comments, particularly for items rated 1 or 2.

Content validity was assessed using the Content Validity Index (CVI) and the Kappa coefficient. The CVI was calculated as the proportion of experts who rated each item as 3 or 4, divided by the total number of experts. Items with a CVI greater than 0.78 were considered excellent, those between 0.50 and 0.78 required revision, and those scoring 0.50 or lower were considered for deletion [35]. The Kappa coefficient was used to estimate the degree of agreement among the experts, with values above 0.75 indicating excellent consensus [35, 36].

All expert feedback was reviewed by the core research team, which consisted of three public health researchers and one statistician. Items were revised or retained based on both the expert panel’s ratings and the team’s consensus discussions.

### Forward-Backward translation

A forward-backward translation process was conducted, involving four independent translators. All were Indonesian nationals enrolled in Malaysian public universities, proficient in both Indonesian and English, culturally familiar with both Malaysia and Indonesia, and experienced in translation work.

The translation process involved the following steps: two translators independently translated the original English version of the SAS into Indonesian (forward translation). The core research team then reviewed and reconciled both versions into a single consensus translation. This version was subsequently back-translated into English by two other independent translators who had not seen the original English version. The core research team compared the back-translated versions with the original to identify and resolve any discrepancies or conceptual deviations. The final Indonesian version of the SAS was approved for pilot testing.

Throughout the process, translators were instructed to prioritise conceptual equivalence over literal translation and to use language that would be easily understood by the target population.

### Pilot testing

This study was conducted in the Klang Valley region of Peninsular Malaysia. Participants were members of *Komunitas Serantau*, an Indonesian migrant workers’ community-based organisation (CBO). The sample included both documented and undocumented migrant workers employed in the manufacturing, construction, plantation, agriculture, or services sectors, except for domestic work. Migrant domestic workers were excluded due to their limited opportunities for social interaction, as they typically reside in their employers’ homes [37]. The sample size was 100, based on minimum sample size recommendations for factor analysis [38, 39].

Recruitment took place between March and May 2024 using a convenience sampling method, with assistance from the leader of *Komunitas Serantau*, who also served as the enumerator. Potential participants were contacted in person, online, or by phone. Each participant received an information sheet detailing the study’s purpose, procedures, voluntary nature, confidentiality protections, and their rights as research participants. Written informed consent was obtained prior to participation. For those with limited literacy, the information was explained orally, and verbal consent was obtained and documented.

Data collection was conducted in small groups at safe and convenient locations, including participants’ homes, the enumerator’s residence, or during community gatherings. A test–retest reliability assessment was conducted with a subset of 30 participants two weeks after the initial administration. Each participant received RM10 (USD 2) as a token of appreciation.

Confidentiality and anonymity were strictly maintained. No personal identifiers, such as names or contact details, were collected. Each participant was instead assigned a numerical code for data entry and management. For the test-retest phase, follow-up was facilitated by the enumerator, without the research team having access to any identifying information.

Descriptive statistics, including frequencies, means, and standard deviations (SD), were calculated using IBM SPSS Statistics (version 29) to summarise participants’ demographic characteristics.

### Psychometric evaluation

Data suitability was assessed using the Kaiser-Meyer-Olkin (KMO) measure of sampling adequacy (acceptable if > 0.60) and Bartlett’s test of sphericity (significant at *p* < 0.05). Exploratory factor analysis (EFA) was conducted in JASP (version 0.19) to examine the underlying structure of the adapted items. Factor extraction was performed using principal axis factoring with varimax rotation. The number of factors to retain was determined using Horn’s parallel analysis, supplemented by visual inspection of the scree plot [40]. Internal consistency was evaluated using Cronbach’s alpha for factors with three or more items [41], and the Spearman-Brown coefficient for two-item factors, with values ≥ 0.70 considered acceptable [42]. Test-retest reliability was assessed using the intraclass correlation coefficient (ICC) to evaluate measurement stability [43].

## Results

### Content validity

The evaluation of the original 12 items of the SAS by the expert panel demonstrated that eight items were rated as having excellent content validity, with CVI scores ranging from 0.80 to 1.00 and Kappa values from 0.76 to 1.00 (Table 1).

**Table 1:** CVI and kappa coefficient for item relevancy and clarity

Item	Relevance		Clarity		Result
	CVI	Kappa	CVI	Kappa	
1	1.00	1.00	1.00	1.00	Validated
2	0.40	0.13	0.60	0.42	Excluded
3	0.80	0.76	1.00	1.00	Validated
4	0.40	0.13	0.80	0.76	Excluded
5	0.80	0.76	0.60	0.42	Validated
6	0.80	0.76	0.80	0.76	Validated
7	0.40	0.13	0.60	0.42	Excluded
8	0.80	0.76	0.80	0.76	Validated
9	0.80	0.76	0.80	0.76	Validated
10	0.80	0.76	0.80	0.76	Validated
11	0.80	0.76	0.80	0.76	Validated
12	0.40	0.13	0.60	0.42	Excluded

Four items (Items 2, 4, 7, and 12) were excluded due to poor relevance and clarity, each receiving a CVI of 0.40 and a Kappa coefficient of 0.13, along with consistent negative qualitative feedback from the expert panel. Item 2 (“What language(s) did you use as a child?“) was deemed unlikely to contribute meaningful data, as the target population is generally known to have limited formal education [23, 33] and to have used only their native dialect during childhood. Item 4 (“In which language(s) do you usually think?“) was considered redundant with Items 1 and 3. For this population, the language spoken at home typically corresponds with the language of thought, rendering the item repetitive. Item 7 (“In what language(s) are the radio programs you usually listen to?“) was viewed as outdated and contextually irrelevant, as radio is not commonly used by the target population in contemporary settings. Item 12 (“If you could choose your children’s friends, you would want them to be”) was considered inapplicable, particularly because migrant workers in Malaysia do not have the legal right to family reunification [44, 45], making the item largely hypothetical.

Revisions were made to Items 6 and 8 to better reflect current media consumption patterns. The original questions—“In what language(s) are the TV programs you usually watch?” and “In general, in what language(s) are the movies, TV, and radio programs you prefer to watch and listen to?”—were revised to “What language do you usually use on social media (such as Facebook, Instagram and TikTok)?” and “In general, what languages are you more comfortable with when using social media (such as Facebook, Instagram and TikTok)?”, respectively.

Wording improvements were also made to other items to enhance clarity, along with adjustments to the scoring scale to account for the multilingual nature of the target population and the diverse social networks of labour migrants. The final questionnaire comprised eight items (Table 2), which were then carried forward for translation.

**Table 2:** Final eight items of the adapted SAS

Item	Description
1	In general, what language do you use to read and speak?
3	What language do you usually use when speaking at home in Malaysia?
5	What language do you usually use to speak with people around you in Malaysia (such as friends, colleagues and employer)?
6	What language do you usually use on social media (such as Facebook, Instagram and TikTok)?
8	In general, what languages are you more comfortable with when using social media (such as Facebook, Instagram and TikTok)?
9	Your close friends are:
10	You prefer going to social gatherings/parties/religious events at which the people are:
11	The persons you visit or who visit you are:

### Translation

The adapted SAS was successfully translated into Indonesian with semantic and conceptual equivalence with the original English version. Minor discrepancies emerged between translators, particularly in terms of formality (e.g., formal vs. informal expressions), sentence structure, and pronoun usage (e.g., first-person vs. second-person). For example, Item 9 was translated in two different ways: *“Siapakah sahabat baik bapak/ibu saat ini?”* (who is your current best friend?*)* and *“Teman dekat saya adalah”* (my close friend is). The consensus version adopted *“Teman-teman dekat anda adalah”* (your close friends are), aligned more closely with the original in both structure and perspective. All discrepancies were resolved through discussion and consensus among the core research team. The final Indonesian version of the adapted 8-item SAS is provided in Supplementary Information (SI) 1.

### Participants’ demographic characteristics

A total of 135 Indonesian migrant workers participated in the pilot testing. The sample consisted of 51.1% male and 48.9% female. Participants ranged in age from 19 to 58 years (mean = 36.29, SD = 10.65). Length of residence in Malaysia ranged from 1 to 35 years (mean = 8.08, SD = 7.23). Reported monthly income ranged from RM1,000 (USD 210) to RM3,000 (USD 630), with a mean of RM1,718.25 (USD 362) (SD = 322.83). The majority were native Javanese speakers (44.8%), had completed secondary education (51.5%), and were employed in the services sector (52.3%) (Table 3).

**Table 3:** Demographic characteristics of the pilot sample (*n* = 135)

Variable		Mean	SD
Age (years)		36.29	10.65
Duration in Malaysia (years)		8.08	7.23
Monthly income (RM)		1,718.25	322.83
		**n**	**%**
Gender	Male	69	51.1
	Female	66	48.9
Native language	Javanese	60	44.8
	Sundanese	8	6.0
	Madurese	14	10.4
	Others	52	38.8
	Missing data	1	-
Education level	No formal education	1	0.7
	Primary education	22	16.4
	Junior high school	41	30.6
	High school/Vocational school	69	51.5
	Diploma	1	0.7
	Missing data	1	-
Employment Sector	Manufacturing	20	15.4
	Construction	25	19.2
	Plantation	11	8.5
	Agriculture	6	4.6
	Services	68	52.3
	Missing data	5	-

### Construct validity

The data met the assumptions for factor analysis, with a KMO value of 0.683 and a significant Bartlett’s test of sphericity (Χ² (28) = 346.058, *p* < 0.001). Horn’s parallel analysis supported the retention of three factors, as the first three observed eigenvalues (2.903, 1.792, and 1.124) exceeded the corresponding simulated mean eigenvalues (0.665, 0.253, and 0.163). The scree plot also supported this three-factor solution and is provided in SI 2. Factor 1 explained 25.6% of the variance and reflected language preferences in media consumption (Items 1, 6, and 8). Factor 2 explained 17.9% of the variance, and related to language use in everyday activities (Items 3 and 5). Factor 3 explained 16.3% of the variance and represented ethnic preferences in social settings (Items 9, 10, and 11). Together, the three factors accounted for 59.8% of the total variance, exceeding the commonly recommended 50% threshold [46] (Table 4).

**Table 4:** Factor structure and psychometric properties of the adapted 8-Item Indonesian version of the SAS

		Factor 1 (Language preferences in media consumption)	Factor 2 (Language use in everyday activities)	Factor 3 (Ethnic preferences in social settings)
Factor loading	Item 8	0.875		
	Item 6	0.829		
	Item 1	0.588		
	Item 5		0.996	
	Item 3		0.592	
	Item 10			0.694
	Item 9			0.619
	Item 11			0.557
Eigenvalue		2.903	1.792	1.124
% of variance		25.6	17.9	16.3

### Construct reliability

Internal consistency was acceptable across the three factors. Factor 1 (language preferences in media consumption) demonstrated good reliability, with a Cronbach’s alpha of 0.816. Factor 2 (language use in everyday activities), consisting of two items, had a Spearman-Brown coefficient of 0.732, indicating acceptable internal consistency. Factor 3 (ethnic preferences in social settings) also showed acceptable reliability, with a Cronbach’s alpha of 0.679 (Table 5) [41, 42].

**Table 5:** Internal consistency of the adapted 8-Item Indonesian version of the SAS

Measure	Factor 1 (Language preferences in media consumption)	Factor 2 (Language use in everyday activities)	Factor 3 (Ethnic preferences in social settings)
Cronbach’s alpha	0.816	–	0.679
Spearman-Brown coefficient	–	0.732	–

Cronbach’s alpha is reported for factors with three or more items. The Spearman–Brown coefficient is used for the two−item factor

Test-retest reliability, based on a subsample of 30 participants, yielded ICC values ranging from 0.991 to 1.000 (*p* < 0.001), indicating excellent temporal stability of the items (Table 6) [43].

**Table 6:** Intraclass correlation coefficients (ICC) for Test-Retest reliability of the adapted 8-Item Indonesian version of the SAS (*n* = 30)

Item	ICC
1	0.991^c^
3	0.991^c^
5	1.000^c^
6	1.000^c^
8	1.000^c^
9	1.000^c^
10	1.000^c^
11	1.000^c^

## Discussion

In this study, we cross-culturally adapted and validated the SAS for use among the Indonesian migrant worker population in Malaysia. The final version consisted of eight items, and was translated into Indonesian through a rigorous forward-backward translation process. Four items were excluded following expert review, as they were deemed culturally and contextually irrelevant for the target population. Specifically, the excluded items did not align with the lived experiences of Indonesian migrant workers in Malaysia—particularly in relation to their background [23, 33], current media consumption habits, and limited opportunities for family reunification [44, 45]. Their exclusion was therefore necessary to preserve the conceptual integrity of the acculturation construct and to ensure that the adapted SAS remains valid, interpretable, and meaningful within this sociocultural context. Retaining culturally irrelevant items could have introduced measurement bias or error, ultimately compromising the accuracy of acculturation assessment. Additionally, two items were modified to reflect the growing use of social media over movies, TV, and radio programs. The scoring scale was also adjusted to account for the multilingual realities of Indonesian migrant workers in Malaysia and their social networks.

Factor analysis revealed that our adapted 8-item Indonesian version of the SAS retained a three-factor structure, consistent with the original study [12] and subsequent validation studies [13, 16, 17]. This supports the structural robustness of the scale across diverse cultural contexts, including among Indonesian migrant workers in Malaysia. However, we observed differences in the order of the factors. Subscale 2 (language preferences in media consumption) emerged as the dominant factor, while Subscale 1 (language use in everyday activities) became secondary. This shift may reflect the increasingly prominent role of media-based language exposure in the acculturation process for Indonesian migrant workers in Malaysia. Rather than relying on face-to-face interactions, this population often engages with Indonesian and Malay content on social platforms to maintain cultural identity while adapting to the linguistic and social norms of Malaysian society. In addition, Item 1, originally part of Subscale 1, was reclassified into Subscale 2. This reclassification may reflect the multilingual realities of this population [47], where code-switching between regional dialects, Indonesian, and Malay is common—particularly in media consumption contexts.

Our adapted 8-item Indonesian version of the SAS demonstrated good internal consistency across all three factors, consistent with the original study [12]. However, a moderately higher internal consistency was observed for Subscale 2 (language preferences in media consumption). This may reflect the population’s linguistic homogeneity in media consumption habits, potentially shaped by their multilingual backgrounds [33, 47, 48]. Furthermore, the high test–retest reliability underscores the temporal stability of the adapted scale.

While these findings support the validity and reliability of the 8-item Indonesian version of the SAS for use among Indonesian migrant workers in Malaysia, several limitations should be acknowledged. First, the relatively small sample size may have limited the ability to conduct confirmatory factor analysis (CFA) to further verify the factor structure identified in the exploratory phase. Second, as with all self-report measures, there is a risk of bias related to social desirability and memory recall, which may have influenced the accuracy of participants’ responses. Third, given the high degree of lexical similarity between Indonesian and Malay [49], language-based items may be less sensitive in capturing distinct differences in linguistic adaptation. Nonetheless, language remains a relevant indicator of acculturation in this context, as the target population is ethnolinguistically diverse, speaking multiple native languages—such as Buginese and Javanese [33, 48]—and the two national languages still differ in vocabulary, spelling, pronunciation, and context-specific usage [49], which may pose challenges when adapting to Malaysia’s linguistic norms.

Future studies should recruit larger and more representative samples to enable CFA and assess the model fit of the adapted instrument. To strengthen the practical utility of the adapted SAS, future research should also aim to establish empirically supported cut-off scores tailored to this population, possibly using classification methods or cluster-based approaches to identify thresholds that meaningfully distinguish levels of acculturation among Indonesian migrant workers.

## Conclusion

This study involved the cross-cultural adaptation and psychometric validation of the SAS for use among Indonesian migrant workers in Malaysia. Through expert panel review and a rigorous translation process, an 8-item version was developed to ensure cultural and contextual relevance for this population. The findings supported the validity and reliability of the adapted 8-item Indonesian version of the SAS in assessing acculturation among Indonesian migrant workers in Malaysia.

## Supplementary Information

Below is the link to the electronic supplementary material.

Supplementary Material 1.

Supplementary Material 2.

## Data Availability

All data relevant to the study are included in the article or provided as supplementary information. Due to ethical constraints and to protect participant confidentiality, the full dataset is not publicly available. However, access to the data may be requested from the Universiti Malaya Research Ethics Committee, Research Services Division, Research and Innovation Management, Universiti Malaya, 50603 Kuala Lumpur Malaysia (email: umrec@um.edu.my), under reference number UM.TNC2/UMREC_3136.
