# Supplementary material for: Cross-cultural adaptation and psychometric properties of the Indonesian version of the short acculturation scale
Source: Discov Psychol. 2025 Sep 30;5(1):96. doi: 10.1007/s44202-025-00429-1 (PMC12484274; doi:10.1007/s44202-025-00429-1)
Supplement: Supplementary file 1 — Supplementary Material 1. [file 44202_2025_429_MOESM1_ESM.docx]

**Cross-cultural adaptation and psychometric properties of the Indonesian version of the Short Acculturation Scale**

**Authors**

Amirah Zafirah Zaini^1^, Mahmoud Danaee^1,^*,Tharani Loganathan^2^, Sally Hargreaves^3^, Hazreen Abdul Majid^4,5^

**Affiliations**

^1^ Department of Social and Preventive Medicine, Faculty of Medicine, Universiti Malaya, 50603 Kuala Lumpur, Malaysia

^2^ Centre for Epidemiology and Evidence-Based Practice, Department of Social and Preventive Medicine, Faculty of Medicine, Universiti Malaya, 50603 Kuala Lumpur, Malaysia

^3^ The Migrant Research Group and The Consortium for Migrant Worker Health, Institute for Infection and Immunity, City St George’s, University of London, London, SW17 0RE, United Kingdom

^4^ School of Health and Rehabilitation Sciences, Health Sciences University, Parkwood Campus, Bournemouth, BH5 2DF, United Kingdom

^5^ Centre for Population Health, Department of Social and Preventive Medicine, Faculty of Medicine, Universiti Malaya, 50603 Kuala Lumpur, Malaysia

******Included:*** *The final Indonesian version of the adapted 8-item Short Acculturation Scale (SAS) is shown here*

**The final Indonesian version of the adapted 8-item Short Acculturation Scale (SAS)**

| *Instruksi: Silahkan jawab pertanyaan di bawah ini dengan lingkari angka 1-5 yang paling menggambarkan jawaban anda.*   1. *Saya* **hanya menggunakan Bahasa Indonesia/Bahasa daerah** 2. *Saya* **menggunakan Bahasa Indonesia/Bahasa daerah lebih baik dari Bahasa Malaysia** 3. *Saya* **menggunakan Bahasa Indonesia/Bahasa daerah dan Bahasa Malaysia kurang lebih sama** **(50:50)** 4. *Saya* **menggunakan Bahasa Malaysia lebih baik dari Bahasa Indonesia/Bahasa daerah** 5. *Saya* **hanya menggunakan Bahasa Malaysia** | | | | | | |
| --- | --- | --- | --- | --- | --- | --- |
|  | Secara umum, bahasa apa yang anda gunakan untuk membaca dan berbicara? | 1 | 2 | 3 | 4 | 5 |
|  | Bahasa apa yang biasanya anda gunakan saat berbicara di rumah di Malaysia? | 1 | 2 | 3 | 4 | 5 |
|  | Bahasa apa yang biasanya anda gunakan untuk berbicara dengan orang-orang sekitar anda di Malaysia (misalnya teman, rekan kerja dan majikan)? | 1 | 2 | 3 | 4 | 5 |
|  | Anda biasanya menggunakan media sosial (*Facebook, Instagram* dan *TikTok*) dengan bahasa apa? | 1 | 2 | 3 | 4 | 5 |
|  | Secara umum, anda lebih nyaman menggunakan media sosial (*Facebook, Instagram* dan *TikTok*) menggunakan bahasa apa? | 1 | 2 | 3 | 4 | 5 |
| *Instruksi: Silahkan jawab pertanyaan di bawah ini dengan lingkari angka 1-5 yang paling menggambarkan jawaban anda:*   1. *Jika jawaban anda adalah* **Semuanya orang Indonesia** 2. *Jika jawaban anda adalah* **Lebih banyak orang Indonesia dari orang Malaysia** 3. *Jika jawaban anda adalah* **Orang Indonesia dan orang Malaysia kurang lebih jumlahnya sama** **(50:50)** 4. *Jika jawaban anda adalah* **Lebih banyak orang Malaysia dari orang Indonesia** 5. *Jika jawaban anda adalah* **Semuanya orang Malaysia** | | | | | | |
|  | Teman-teman dekat anda adalah: | 1 | 2 | 3 | 4 | 5 |
|  | Anda lebih menyukai untuk datang ke acara syukuran/arisan/pengajian yang pesertanya adalah: | 1 | 2 | 3 | 4 | 5 |
|  | Orang-orang yang anda kunjungi atau mengunjungi anda adalah: | 1 | 2 | 3 | 4 | 5 |
