# Supplementary material for: Cross-cultural adaptation and psychometric properties of the Indonesian version of the short acculturation scale
Source: Discov Psychol. 2025 Sep 30;5(1):96. doi: 10.1007/s44202-025-00429-1 (PMC12484274; doi:10.1007/s44202-025-00429-1)
Supplement: Supplementary file 2 — Supplementary Material 2. [file 44202_2025_429_MOESM2_ESM.docx]

**Cross-cultural adaptation and psychometric properties of the Indonesian version of the Short Acculturation Scale**

**Authors**

Amirah Zafirah Zaini^1^, Mahmoud Danaee^1,^*,Tharani Loganathan^2^, Sally Hargreaves^3^, Hazreen Abdul Majid^4,5^

**Affiliations**

^1^ Department of Social and Preventive Medicine, Faculty of Medicine, Universiti Malaya, 50603 Kuala Lumpur, Malaysia

^2^ Centre for Epidemiology and Evidence-Based Practice, Department of Social and Preventive Medicine, Faculty of Medicine, Universiti Malaya, 50603 Kuala Lumpur, Malaysia

^3^ The Migrant Research Group and The Consortium for Migrant Worker Health, Institute for Infection and Immunity, City St George’s, University of London, London, SW17 0RE, United Kingdom

^4^ School of Health and Rehabilitation Sciences, Health Sciences University, Parkwood Campus, Bournemouth, BH5 2DF, United Kingdom

^5^ Centre for Population Health, Department of Social and Preventive Medicine, Faculty of Medicine, Universiti Malaya, 50603 Kuala Lumpur, Malaysia

******Included:*** *Scree plot for parallel analysis of the* *Indonesian version of the adapted 8-item Short Acculturation Scale (SAS) is shown here*

**Scree plot for parallel analysis of the Indonesian version of the adapted 8-item Short Acculturation Scale (SAS)**


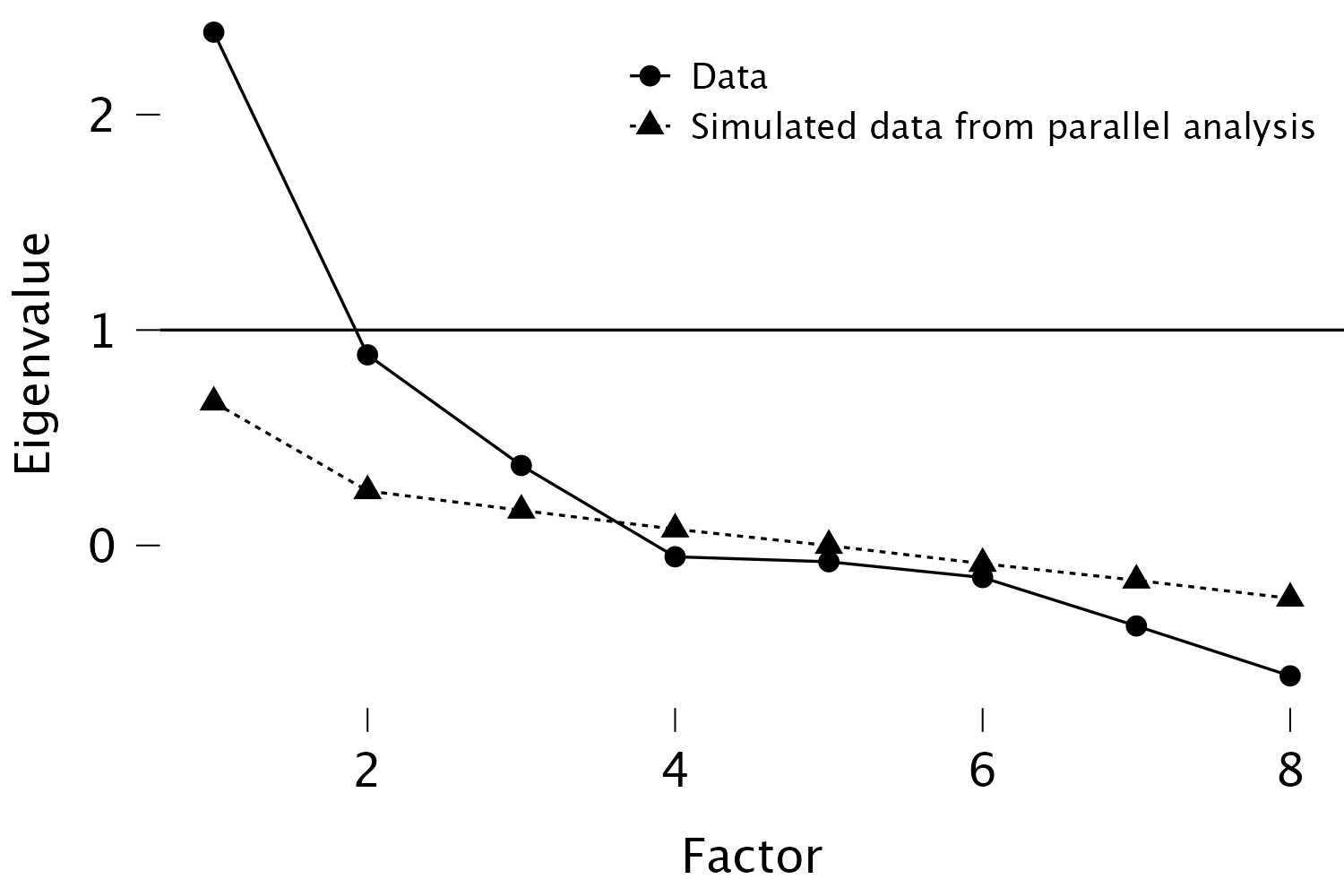


The scree plot indicated the retention of three factors, as the eigenvalues of the first three components exceeded the corresponding eigenvalues from the simulated data, confirming the presence of three meaningful factors among the eight items.
